# Inhibitory Effect of Sophorolipid on *Candida albicans* Biofilm Formation and Hyphal Growth

**DOI:** 10.1038/srep23575

**Published:** 2016-03-31

**Authors:** Farazul Haque, Md. Alfatah, K. Ganesan, Mani Shankar Bhattacharyya

**Affiliations:** 1 Biocatalysis and Fermentation Science Laboratory, Biochemical Engineering Research & Process Development Center (BERPDC), CSIR-Institute of Microbial Technology, Sector 39-A, Chandigarh-160 036, India; 2 Yeast Molecular Biology Laboratory, CSIR-Institute of Microbial Technology, Sector 39-A, Chandigarh-160 036, India

## Abstract

*Candida albicans* causes superficial and life-threatening systemic infections. These are difficult to treat often due to drug resistance, particularly because *C. albicans* biofilms are inherently resistant to most antifungals. Sophorolipid (SL), a glycolipid biosurfactant, has been shown to have antimicrobial and anticancer properties. In this study, we investigated the effect of SL on *C. albicans* biofilm formation and preformed biofilms. SL was found to inhibit *C. albicans* biofilm formation as well as reduce the viability of preformed biofilms. Moreover, SL, when used along with amphotericin B (AmB) or fluconazole (FLZ), was found to act synergistically against biofilm formation and preformed biofilms. Effect of SL on *C. albicans* biofilm formation was further visualized by scanning electron microscopy (SEM) and confocal laser scanning microscopy (CLSM), which revealed absence of hyphae, typical biofilm architecture and alteration in the morphology of biofilm cells. We also found that SL downregulates the expression of hypha specific genes *HWP1*, *ALS1*, *ALS3*, *ECE1* and *SAP4*, which possibly explains the inhibitory effect of SL on hyphae and biofilm formation.

Candidiasis caused by *Candida* species is one of the most common form of hospital acquired opportunistic infection^1^,^2^. Though *C. albicans* remains the major causative agent, infection caused by other *Candida* species like *C. tropicalis, C. glabrata*, *C. lusitaniae, C. parapsilosis* and *C. krusei* are becoming more prevalent^1^,^3^,^4^,^5^. Immunocompromised patients and patients with medically implanted devices (catheters, heart valves, cardiac pacemakers, vascular bypass grafts, endotracheal tubes and central nervous system shunts) are highly susceptible to *Candida* infections^6^,^7^,^8^. Despite the use of antifungal therapies, due to delayed diagnosis and antifungal resistance, candidiasis is associated with high mortality worldwide^2^,^9^,^10^. An important reason for the failure of current antifungal drugs is attributed to *Candida* biofilms which are inherently resistant to most antifungal treatments. Biofilm is an organized community of cells, embedded in a matrix of exopolymeric substances^7^,^11^,^12^. Adherence and colonization of planktonic cells on host tissues and medical devices initiates formation of biofilms^12^,^13^,^14^. The most notorious feature of biofilms is its several fold higher resistance to antifungal drugs compared to their planktonic counterparts^8^,^15^,^16^. Moreover, few antifungal drugs which are currently used in treatment, have other limitations such as severe toxicity^17^,^18^,^19^. Thus, there is an urgent need for newer antifungal drugs that are potentially active alone or in combination with current antifungals against both the planktonic cells and biofilm of *Candida*

Biosurfactants show antiadhesive and antimicrobial activities^20^,^21^,^22^. Sophorolipid (SL) is a glycolipid biosurfactant, produced by several *Starmerella* species^23^,^24^,^25^. Naturally synthesized SL is a mixture of acidic and lactonic forms and their abundance depends on the producer species^26^. SL exhibits low cytotoxicity and its use in food and pharmaceutical industries have been approved by US FDA^27^. Lactonic form of SL has antimicrobial and anticancerous properties^23^,^25^,^28^. Antifungal activity of SL against planktonic cells of pathogenic *Candida* species has also been reported. However, the activity of SL against *Candida* biofilms is not known. Recent reports showed that combinatorial therapy of various drugs is highly effective to eradicate *Candida* biofilm^29^. In fact, combinatorial therapy against pathogens has several advantages which includes rapid effect of the therapy, wide drug spectrum, synergy, lowered toxicity and lowered risk for antifungal resistance. In the present study we investigated the effect of SL on *Candida* biofilm formation and preformed biofilms of *Candida*, alone and in combination with AmB or FLZ. We have also investigated the mechanistic basis for SL mediated biofilm inhibition, which is possibly through inhibitory effect of SL on hyphae formation. Hyphae are the one of the major constituents of biofilms.

## Results

### SL showed antifungal activity against both *Candida albicans* and non-*albicans Candida* (NAC) strains

The MIC (minimum inhibitory concentration) for SL was determined against *C. albicans* and NAC in RPMI-1640 medium employing standard CLSI method^30^. Planktonic cells of *C. albicans* were incubated with serially double-diluted concentrations of purified SL (0–1920 μg/ml) in 96-well microtiter plates and incubated at 37 °C for 2 days. At the end of incubation, growth of cells was determined by OD_600_ _nm_ reading. The MIC_80_ is defined as the lowest concentration of SL which inhibits 80% cell growth as compared to control (without SL). MIC_80_ of *C. albicans* was found to be 60 μg/ml (Table 1). Higher concentration led to the complete inhibition of growth. In an attempt to find out the effect of SL on non-*albicans Candida* (NAC) species, we extended our study to *C. lusitaniae, C. tropicalis* and *C. glabrata*. The MIC_80_ for *C. tropicalis* and *C. glabrata* was 60 μg/ml and 120 μg/ml, respectively. *C. lusitaniae* was found to be the most susceptible (MIC80 30 μg/ml) among NAC strains tested (Table 1).

### SL inhibits biofilm formation and eradicates the preformed biofilm

Activity of SL was tested on bioflm formation of *C. albicans* and NAC strains. Biofilm formation was initiated in 96-well microtiter plates in the presence of serially double diluted concentrations of SL (0–1920 μg/ml) and incubated at 37 °C for 2 days. Quantification of biofilms was performed by colorimetric XTT reduction assay and viability was expressed in terms of percentage metabolic activity. The BIC_80_ (biofilm inhibiting concentration) was defined as the lowest concentration of SL that inhibits 80% metabolic activity of biofilm formation as compared to control (without SL). We found that *C. glabrata* has highest BIC_80_ (480 μg/ml) (Table 1, Supplementary Fig. S1), whereas, BIC_80_ for *C. albicans, C. tropicalis* and *C. lusitaniae* was 120 μg/ml (Table 1; Supplementary Fig. S1).

**Figure 1:**
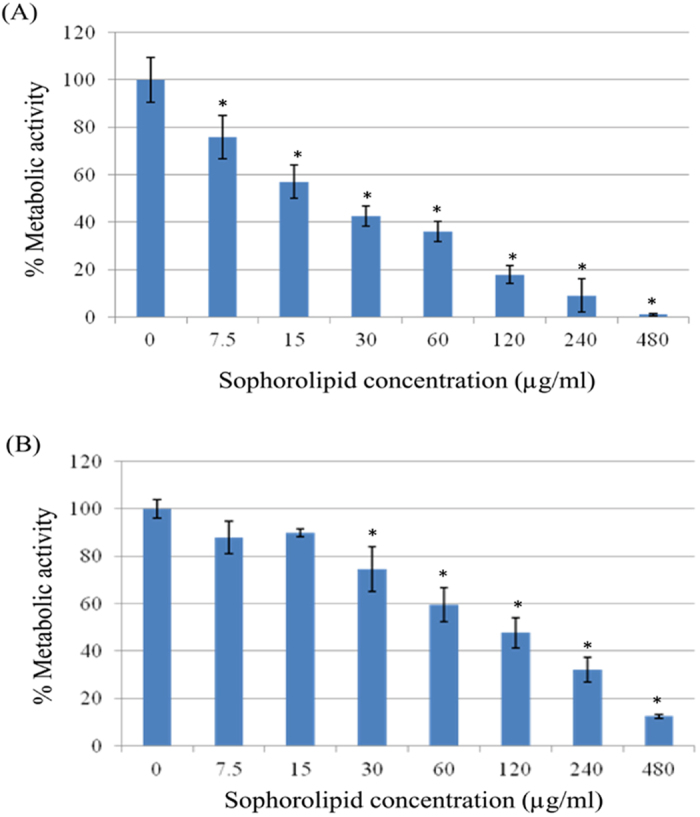
Effect of sophorolipid on *C. albicans* biofilm formation (A) and preformed biofilms (B). Readings of colorimetric XTT reduction assay at 492 nm are expressed in terms of % metabolic activity of control. BIC_80_ and BEC_80_ of SL against biofilm formation and preformed biofilms, respectively, are defined as the minimum concentration of SL at which 80% reduction in the metabolic activity of biofilm is seen as compared to the control. Results represent the average of three independent experiments ± SD. ^*^p < 0.05 when compared with the SL untreated controls.

To know the antifungal efficacy of SL against *C. albicans* and NAC strain mature biofilms, we performed SL susceptibility testing against preformed biofilms. Biofilms were formed in 96-well microtiter plates for 2 days at 37 °C and thereafter, serially double-diluted concentrations of SL (0–1920 μg/ml) were added to preformed biofilms and further incubated at 37 °C for 2 days. Subsequently, metabolic activity was determined by colorimetric XTT reduction assay. The BEC_80_ (biofilm-eradicating concentration) was defined as the lowest concentration of SL that eradicates 80% of biofilm compared to conrol (SL untreated biofilms). The BEC_80_ for *C. albicans* was 4-fold higher (480 μg/ml) compared to biofilm forming planktonic cells (BIC_80_ 120 μg/ml) (Table 1). Viability of preformed biofilms was found to be inhibited by SL in a concentration dependent manner (Fig. 1B). For *C. tropicalis* BEC_80_ was 480 μg/ml whereas, *C.glabrata* was found to be highly resistant to SL, followed by *C. lusitaniae* where BEC_80_ was not determined in both cases (Table 1; Supplementary Fig. S2).

### SL affectcs biofilm cells morphology

**Figure 2:**
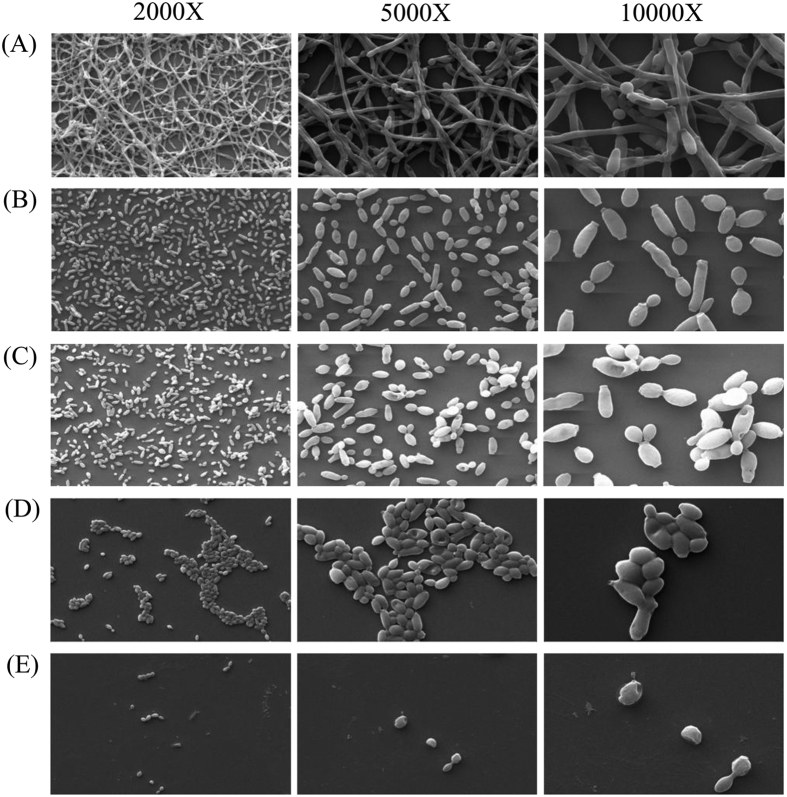
Scanning electron microscopy images of *C. albicans* biofilms. Effect of sophorolipid on *C. albicans* biofilm formation was analyzed by SEM at indicated magnifications. Biofilms were formed on coated poly-L-lysine glass cover slips in 6-well cell culture plates at 37 °C for 2 days in the presence of 0 μg/ml (**A**), 60 μg/ml (**B**), 120 μg/ml (**C**), 240 μg/ml (**D**) and 480 μg/ml (**E**) of SL.

**Figure 3:**
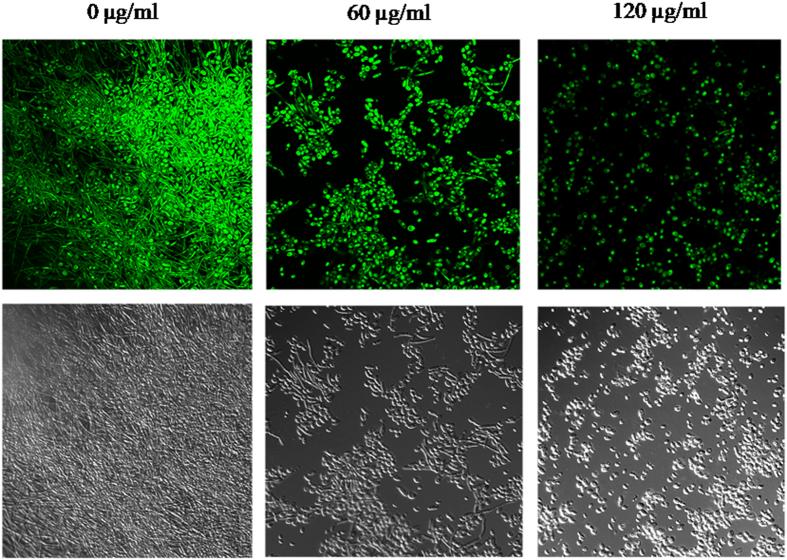
Confocal laser scanning microscopy images of *C. albicans* biofilms. Biofilms were formed at the indicated concentrations of sophorolipid on coated poly-L-lysine glass cover slips in 6-well cell culture plates at 37 °C for 2 days. Biofilms were stained with SYTO 9 (green fluorescence) and visualized at 60X magnification (upper panel). DIC of respective images is shown in the lower panel.

We further explored the effect of SL on *C. albicans* biofilm and their cellular morphology. Biofilms were formed in presence of serially double-diluted concentrations of SL (0–1920 μg/ml) on poly-L-lysine coated glass cover slips in 6-well microtiter plates for 2 days at 37 °C, and visualised by SEM and CLSM. SEM images of control sample (0 μg/ml SL) demonstrated the presence of complex structure of biofilm having hyphae and yeast cells (Fig. 2A). Biofilms formed in the presence of 60 μg/ml SL was devoid of hyphal organization and consisted mostly of yeast cells (Fig. 2B). It is worth noting that at this concentration biofilm formation was reduced only by 60% (Fig. 1A), indicating that SL inhibits hyphal growth even at a lower concentration. At BIC_80_ concentration of SL (120 μg/ml) (Fig. 1A), biofilm cells were found to have perforated outer membrane with swollen and deformed morphology (Fig. 2C). Aggregated population of cells with wrinkled surface can be seen at 240 μg/ml and 480 μg/ml SL concentration respectively (Fig. 2D,E). CLSM image (Fig. 3) showed dense and compact hyphal mass in the control sample (0 μg/ml SL). However, at 60 μg/ml SL concentration yeast cells were more prevalent. Further increase in SL concentration (120 μg/ml) led to the complete inhibition of biofilm and cells remains in the yeast form.

### SL inhibits *Candida albicans* hyphal growth

**Figure 4:**
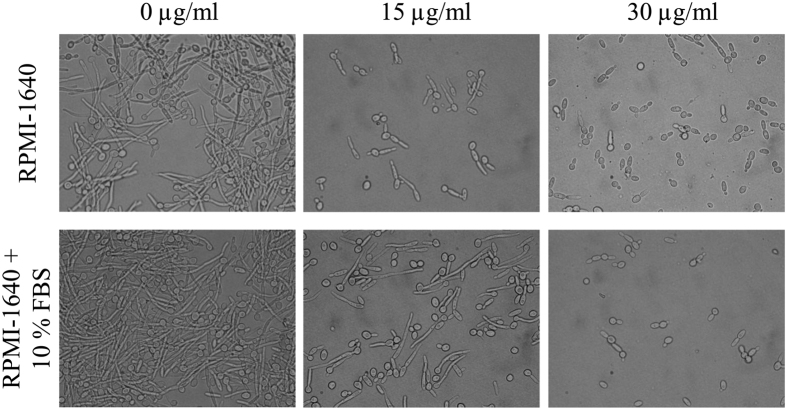
Effect of sophorolipid on *C. albicans* hyphal growth. *C. albicans* cells were grown in RPMI-1640 medium (upper panel) and RPMI-1640 containing 10% FBS (lower panel) at the indicated concentration of SL at 37 °C for 5 hrs. At the end of incubation an aliquot was withdrawn from each sample and photographed at 100× magnification.

**Figure 5:**
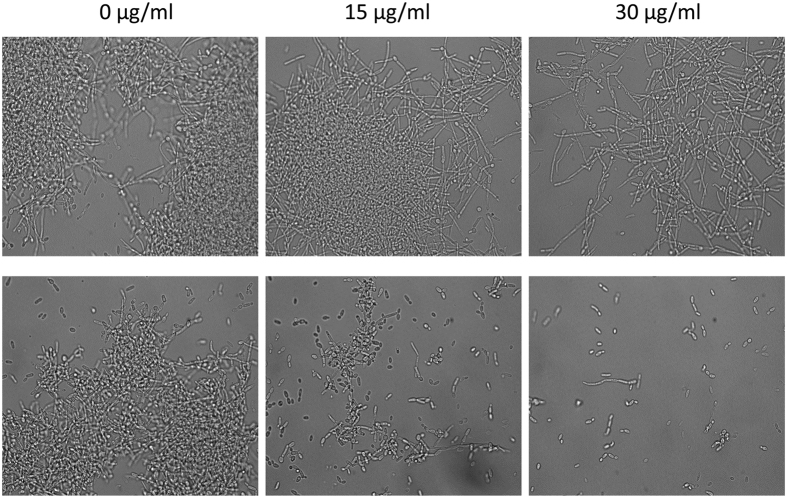
Effect of sophorolipid on *C. albicans* mature hyphae. *C. albicans* cells were grown in RPMI-1640 containing 10% FBS for 5 hrs. After that mature hypha were treated with indicated concentrations of SL for time point zero (upper panel) and 5 hrs (lower panel) at 37 °C. At the end of incubation an aliquot was withdrawn from each sample and photographed at 60× magnification.

We further examined the effect of SL on hyphal growth. *C. albicans* hyphal growth assay was performed in presence of different concentration of SL in RPMI-1640 medium and RPMI-1640 medium containing hypha inducer (10% FBS) at 37 °C. After 5 hrs of incubation aliquots of the cells were microscopically visualized. In control samples (0 μg/ml SL) massive *C. albicans* hyphae were observed (Fig. 4, Supplementary Fig. S3), however hyphal growth was modest at 15 μg/ml SL, while, hyphal growth was absent at 30 μg/ml SL in both media (Fig. 4), indicating concentration dependent inhibition of hyphal growth by SL. Nevertheless, at this concentration of SL (15 μg/ml), growth of hyphae in RPMI-1640 + 10% FBS medium was slightly higher compared to RPMI-1640 medium (Fig. 4), which could be due the effect of hypha inducing supplement (10% FBS). Effect of SL was also examined on mature hyphae of *C. albicans*. The cells were first grown in RPMI 1640 + 10% FBS for 5 hrs at 37 °C and then treated with SL. Untreated sample (0 μg/ml SL) was found to have massive hyphae (Fig. 5). However upon SL (15 μg/ml) treatment, hyphae were shortend as compared to untreated hyphae. Moreover at 30 μg/ml SL concentration cells were completley devoid of hyphae and remains in yeast form.

### SL downregulates *Candida albicans* hyphal specific genes

**Figure 6:**
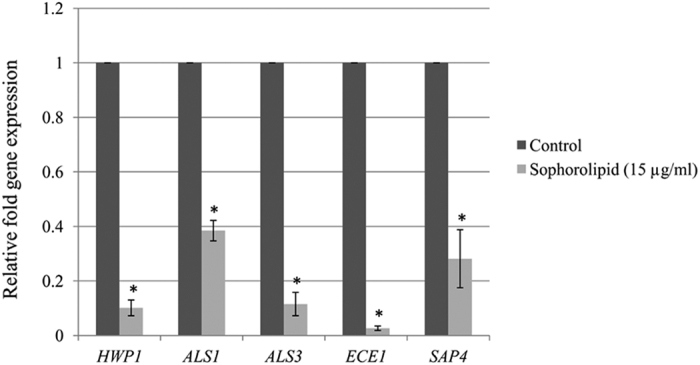
Effect of sophorolipid on the expression of *C. albicans* hypha specific genes. *C. albicans* cells were incubated in the absence (control) or presence (15 μg/ml) of SL in RPMI-1640 medium at 37 °C for 5 hrs. Following incubation expression of the indicated genes were determined by qRT-PCR. Expression level of each gene is displayed after normalization with internal control housekeeping gene *ACT1*. The histogram shows the relative expression fold change of genes by SL treatment with respect to the control. Results represent the average of three independent experiments ± SD. ^*^p < 0.05 when compared with the SL untreated controls.

To gain further insight into the mechanism of SL mediated inhibition of *C. albicans* hyphal growth, we analyzed the expression profile of important hyphal growth associated genes such as *HWP1*, *ALS1*, *ALS3*, *ECE1* and *SAP4*^12^,^31^. Hwp1 (hyphal wall protein 1), Als1 (agglutinin-like sequence 1) and Als3 (agglutinin-like sequence 3) proteins are involved in the maintaining of cell wall integrity and hypha initiation^12^,^31^. Ece1 (extent of cell elongation 1) protein is essential for hypha initiation and elongation^12^,^31^. *SAP4* encodes secreted aspartyl protease 4 protein and its expression is enhanced during yeast to hyphal cells transition^31^,^32^. To test whether SL reduces the expression of these genes resulting in hyphal growth inhibition, we extracted total RNA from cells treated with SL (15 μg/ml) and control (0 μg/ml SL) in RPMI-1640 medium. Transcript levels in SL treated and untreated cells were quantified by qRT-PCR. Expression level of each gene was normalized with housekeeping gene (*ACT1*) for both SL treated as well as untreated cells and presented in the form of relative expression fold change. Expression of *HWP1*, *ALS1*, *ALS3*, *ECE1* and *SAP4* in SL treated cells was reduced significantly by 10-fold, 2.5-fold, 8.7-fold, 37.7-fold and 3.6-fold respectively as compared to control (Fig. 6).

### SL synergistically interacts with AmB and FLZ on C*andida albicans* biofilm formation and preformed biofilms

Interaction of SL with two potent antifungal drugs AmB and FLZ was tested by chequerboard assay on *C. albicans* biofilm formation and preformed biofilms. The predetermined BIC_80_ of SL, AmB and FLZ were 120 μg/ml, 0.25 μg/ml and 256 μg/ml, respectively. The predetermined BEC_80_ of SL and AmB were 480 μg/ml and 4 μg/ml, respectively. However, BEC_80_ of FLZ was not achieved at the highest concentration (1024 μg/ml) used in this study. Fractional inhibitory concentration (FIC) of each compound in each combination (SL and AmB or SL and FLZ) was calculated for biofilm formation and preformed biofilms. SL at 0.125× BIC_80_ and 0.250× BIC_80_ concentrations reduced the BIC_80_ of AmB by 4-fold and of FLZ by 32-fold, respectively (Table 2). Moreover, SL at 0.250× BEC_80_ concentration reduced the BEC_80_ of AmB and FLZ by 8-fold and more than 8-fold, respectively (Table 2). After FIC determination, Fractional inhibitory concentration index (FICI) was calculated for each combination to determine the interaction of SL with AmB or FLZ. The FICI of SL in combination of AmB and FLZ were 0.375 and 0.281, respectively, on biofilm formation, and 0.375 and ≤0.375, respectively, on preformed biofilms (Table 2). These values are ≤0.5, which indicate that SL has synergistic interaction with AmB and FLZ on both biofilm formation and preformed biofilms.

## Discussion

SL is known to have antifungal activity, however, only percent inhibition with a single concentration of SL have been reported. In the present study, we have determined the MIC_80_ of SL against the planktonic cell of *C. albicans, C. tropicalis, C. glabrata and C. lusitania*e (Table 1). Previously, numerous studies have shown that biosurfactants inhibit biofilm formation by preventing adhesion of microorganism to the solid surfaces^20^,^22^,^33^,^34^. Being biosurfactant in nature and having antifungal property, we investigated the effect of SL on *C. albicans* biofilm formation. BEC_80_ of SL was found different in different species. For *C. albicans* and *C. tropicalis* the BEC_80_ was found to be 4- fold higher in concentration as compared to the MIC_80,_ indicating that matured biofilm is moderately resistant towards SL as compared to planktonic cells. During the course of our studies, Mukherji *et al*.^35^ reported the antibiofilm activity of SL against *Vibrio cholera*, indicating that the biofilm inhibitory activity of SL is likely to be broad spectrum. Since *C. albicans* is the major disease causative agent, we further pursued our study on *C. albicans*.

SEM and CLSM analysis of the *C. albicans* biofilm demonstrated the presence of dense hyphae in absence of SL. However, the SEM images at BIC_80_ (120 μg/ml, Fig. 2C) showed deformed and swollen cells with perforated outer membrane. These morphological alterations of the cells could be associated with loss of cell membrane integrity resulting in cell death as reported previously for tetracycline-SL or cefaclor-SL combination treatment against *Staphylococcus aureus* and *Escherichia coli*, respectively^27^. Moreover, deformation of the cells and loss of cell membrane integrity have been reported as the mechanisms of antimicrobial activity for many biosurfactants^36^. Basak *et al*. reported that SL capped ZnO nanoparticle mediated *C. albicans* cell death occurs via membrane bursting followed by oozing out of proteins and intracellular materials^37^. The same phenomena thought to be responsible for SL mediated cell death. Aggregated scant population of biofilm cells with wrinkled surface can be observed at 240 μg/ml and 480 μg/ml of SL concentrations respectively (Fig. 2D,E), indicating complete absence of biofilm formation.

Since hyphal growth is a virulence factor *in C. albicans* infection^31^, inhibition of hyphal growth by SL (Figs 2B and 3) is a significant finding. Besides their role in biofilm formation, hyphae mediate dissemination of *C. albicans* to the host tissues by invasion^31^. It has been reported that virulence of *C. albicans* is reduced in hypha deficient mutants^38^, emphasizing the importance of hypha formation in *C. albicans* infection. The inhibition of hyphal growth at 30 μg/ml concentration of SL, even in the presence of hypha inducing agents (10% FBS) indicated significant role of SL in hyphal growth inhibition.

The effect of subinhibitory concentration of SL on mature hyphae of C. albicans cells were also tested. Hyphae in presence of 15 μg/ml were shortened and completely absent at 30 μg/ml concentration of SL used. There was no traces of broken hypha found in both concentrations (Fig. 5). Another reason for shortening of the hypahe may be due to the morphological plasticity as C. albicans have a capability to undergo reversible morphological changes between yeast, pseudohyphae and hyphal forms in response to environmental stress^41^. Similar results for reverse morphogenesis was observed with gymnemic acid a triterpinoid saponin family compound which, transforms the hyphal cells into yeast form^42^.

To gain insight into the molecular mechanism of SL mediated hyphal growth inhibition, expression profile of hyphal growth associated genes were analyzed. Transcripts result reveal that SL downregulates the expression of hyphal genes resulting in inhibition of hyphal growth. It was earlier reported that *C. albicans* mutants of *HWP1* and *ALS3* are defective in biofilm formation^39^,^40^. Inhibition of expression of these genes by SL (15 μg/ml) (Fig. 6) is consistent with its effect on biofilm formation. At this concentration of SL, metabolic activity of biofilm formation was around 55% as compared to the control (Fig. 1). Transcripts level of *HWP1* and *ALS3* in cells treated with higher concentration of SL was also quantified and found to be further reduced (data not shown). SL mediated down-regulation of the expression of these genes and inhibition of hyphal growth could be a reason for abrogation of *C. albicans* biofilm formation.

*C. albicans* biofilms are intrinsically resistant to most of the current antifungal drugs^19^. High dose antifungal drug therapy against biofilms is always associated with severe side effects^17^,^18^,^19^. Echinocandins have shown some effectiveness against biofilms^43^,^44^,^45^, but recent studies reported that resistance against it is emerging^46^,^47^. Combination therapy is an option to minimize the side effect of existing potent antifungals with the use of less or non-toxic new antifungals to eradicate the *Candida* biofilm and thereby candidiasis infection^48^. Uppuluri and coworkers demonstrated that calcineurin inhibitors FK506 and cyclosporine A in combination with FLZ can work in synergy against *C. albicans* biofilm^49^. Here, we found that, SL inhibits hyphal growth and biofilm formation, reduces the viability of preformed biofilms, and synergistically interacts with antifungal drugs AmB and FLZ in biofilm conditions. Therefore, it could be a potential compound against *Candida* biofilm as well as can be used in combination with AmB and FLZ. To the best of our knowledge, this is the first study demonstrating the role of SL in inhibition of *C. albicans* biofilm formation and hyphal growth. Further evaluation is required to determine the antibiofilm activity of SL *in vivo*. SL also enhances the efficacy of AmB and FLZ against *C. albicans* biofilm, implying a promising synergistic combination for the treatment of candidiasis.

## Methods

### Organisms, media and growth conditions

Wild type strains of *C. albicans* SC5314^18^, *C. glabrata* CG462^18^, *C. tropicalis MYA3404*^50^* and C. lusitaniae* CL6^18^, were used in this study. Frozen glycerol stock of the strain was regularly revived on YPD agar medium (1% Bacto yeast extract, 2% Bacto peptone, 2% glucose and 2% Bacto agar). For broth culture, strain was grown in YPD medium at 30 °C with agitation (200 rpm). RPMI-1640 medium with L-glutamine without sodium bicarbonate (Sigma) was buffered with 0.165 M morpholinepropanesulfonic acid (Sigma) to a pH of 7. Stock solutions of extracted SL (supplementary material), AmB (Sigma) and FLZ (Sigma) were prepared in dimethyl sulfoxide (DMSO, Sigma), and stored at −20 °C until use.

### Purification and characterization of SL

*Starmerella bombicola* MTCC1910, was used for SL production. It was grown as described in the supplement. SL was separated from the fermentation broth by ethyl acetate extraction and concentrated by vacuum evaporation of the solvent at 40 °C. Residual hydrophobic components were washed with n-hexanes to obtain a crude mixture of SL. Different components of the crude mixture were monitored by thin layer chromatography (TLC) (Supplementary Fig. 4) on Merck silica Gel 60 F_254_ 10 cm × 5 cm TLC plates using chloroform /methanol (65:15:2) as mobile phase. Crude mixture of SL was also characterized by HPLC (Shimadzu) with UV detector (207 nm) and a RP-C_18_ column (Merck, 5 μ, 4.5 × 250 mm) using gradient elution. Initially, acetonitrile:water (30:70) was used for 5 min, increased to acetonitrile: water (80:20) in 25 min and maintained there for next 25 min. The flow rate was 0.5 ml/min and injection volume was 10 μl. Column chromatography was carried out to isolate the lactonic form of SL. 50 gm of silica mesh size (60–120) in hexane was packed in (50 × 5 cm) glass column. 200 ml of eluent (chloroform/ methanol) is run through the column before loading the crude SL. 300–400 mg of crude SL dissolved in a small volume of ethanol was mixed with silica (3.5 gm) and evaporated under reduced pressure at 40 °C. Once the silica is fully dried it was loaded into the column. Diacetylaed form of lactonic SL was eluted from the column by using chloroform and methanol at a ratio of 98:2 and dried under vacuum at 40 °C and stored for further use. HPLC analysis showed a single peak of SL with more than 99% purity (Supplementary Fig. 5B). Different functional groups present in the sample were identified by FT-IR spectroscopy (Supplementary Fig. 6) (Bruker optics, vortex 70) confirming the presence of lactonic form of SL in the sample. This preparation of SL was used for determining the anticandida activity in subsequent experiments^51^.

### SL susceptibility testing

SL activity against planktonic cells of *Candida* strains was tested by broth microdilution method using CLSI (Clinical and Laboratory Standards Institute) guidelines^30^. Serially double-diluted concentrations of SL were prepared in RPMI-1640 medium, such that the final concentration of DMSO does not exceed 5% in any assay. 100 μl of each dilution was dispensed into the well of a presterilized, flat-bottomed 96-well polystyrene microtiter plate (Becton Dickinson). RPMI-1640 medium containing 5% DMSO was included in control wells. Planktonic cells grown to exponential phase in YPD broth was harvested, washed with sterile 1X phosphate-buffered saline (PBS) and resuspended in RPMI-1640 medium at a density of 4 × 10^3^ cells/ml. 100 μl of cell suspension was added into the SL containing and control wells to provide 2 × 10^3^ cells/ml in 200 μl working volume. Thereafter, microtiter plates were incubated at 37 °C for 2 days. After incubation, growth of cells was measured by microtiter plate reader (BioTek) at 600 nm.

### Effect of SL on *Candidas* biofilm formation and preformed biofilms

The biofilm formation assay was performed in 96-well microtiter plates as described previously^52^,^53^, with slight modifications. Briefly, the cell suspension was prepared in RPMI-1640 medium at a density of 2 × 10^6^ cells/ml and dispensed into the wells of microtiter plates (100 μl per well). Serially double-diluted concentrations of SL in RPMI-1640 medium were added (100 μl per well) to the wells such that final cell density remains 1 × 10^6^ cells/ml for biofilm formation^53^. Similarly, 100 μl of RPMI-1640 medium containing 5% DMSO without SL was added into the selected wells for control. Microtiter plates were incubated at 37 °C for 2 days.

For preformed biofilms, the cell suspension was prepared in RPMI-1640 medium at a cell density of 1 × 10^6^ cells/ml^52^,^53^. 100 μl of cell suspension was dispensed into the wells of microtiter plates and incubated at 37 °C for 2 days. At the end of incubation medium was aspirated from the wells and nonadherent cells were removed by washing the biofilms 3-times with sterile PBS. Residual PBS of the wells was removed by blotting with paper towels at the end of washing steps. 100 μl of serially double-diluted concentrations of SL were added into the wells of prewashed biofilms. For control, 100 μl of RPMI-1640 medium containing final 5% DMSO without SL was added into the selected wells of biofilms. Further, microtiter plates were incubated at 37 °C for 2 days. The metabolic activity of biofilms was quantitatively determined by colorimetric XTT [2,3-bis(2-methoxy-4-nitro-5-sulfophenyl)-2H-tetrazolium- 5-carboxanilide sodium salt] reduction assay.

### Colorimetric XTT reduction assay

Subsequent to the appropriate incubation of the microtiter plates, medium was aspirated from the wells and nonadherent cells were removed by washing the biofilms as described above. Colorimetric XTT reduction assay of biofilm was performed as previously reported^52^,^53^. 0.5 gm/L stock solution of XTT tetrazolium salt (Sigma) in PBS was filter sterilized through 0.22 μm pore size filter and stored in aliquots at −80 °C. Just prior assay, an aliquot was thawed and 1 μM final concentration of freshly prepared menadione (Sigma) was added to the XTT solution. Hundred (100) μl of XTT-menadione solution was distributed into the wells containing prewashed biofilms and to the empty wells (for the background values of XTT reduction) and incubated at 37 °C in the dark for 1 hr. Colorimetric change in the XTT reduction (reduced formazan-coloured product formation which is correlated with the metabolic activity of the biofilm) was measured in a microtiter plate reader at 492 nm.

### SEM and CLSM analysis of *Candida albicans* biofilm

The effect of SL on biofilms was qualitatively analyzed by SEM and CLSM. Biofilms were formed on poly-L-lysine (Sigma) coated glass cover slips (Blue Star) in 6-well cell culture plates (Nunc). Glass cover slips were coated with poly-L-lysine (2% wt/vol) as described by Dong *et al*.^54^. Coated cover slips were further sterilized by UV radiation for 1 hr under laminar air flow and placed into the wells of microtiter plates for the initiation of biofilms. Biofilms were formed in the presence of serially double-diluted concentrations of SL at 37 °C for 2 days. RPMI-1640 medium containing 5% DMSO without SL was included as control. At the end of incubation cover slips were transferred to new 6-well plates and washed 3-times with PBS.

For SEM, biofilms were dried and processed as described by Ramage *et al*.^55^, with slight modifications. Briefly, PBS washed biofilms were fixed subsequently for 20 min by formaldehyde (4% vol/vol) and glutraldehyde (2% vol/vol), followed by dehydration in a series of ethanol solutions^55^. Final dehydration was carried out by t-butyl alcohol for 30 min at room temperature and then dried in a desiccator. Thereafter, samples were coated with gold palladium for 135 sec at 10–12 milli amperes current and visualized by scanning electron microscope (ZEISS EVO 40) in high-vacuum mode at 20 kV.

For CLSM, biofilms were stained as described previously^56^ with fluorescent stains SYTO 9 (Molecular Probes) which stain live cells. Coverslips containing biofilms were incubated with 6.6 μM final concentration SYTO 9 for 30 min in dark. Following incubation slides were visualized with the Nikon A1R confocal microscope using 60X objective lens. Images were analyzed with NIS Elements software.

### Effect of SL on *Candida albicans* hyphal growth

Hyphal growth assay was performed in 10 ml of RPMI-1640 medium and RPMI-1640 medium supplemented with 10% foetal bovine serum (FBS, Invitrogen). Cell suspension was diluted at 1 × 10^7^ cells/ml in medium and incubated with different concentrations of SL (0 μg/ml, 15 μg/ml and 30 μg/ml) at 37 °C with agitation (200 rpm) for 5 hrs. Aliquots of samples were visualized under bright field using 100X objective lens by Zeiss fluorescence microscope and photographed. Samples incubated in different concentrations of SL for zero hour were also examined under similar condition.

### Effect of SL on *Candida albicans* mature hypha

Effect of SL on *C. albicans* hyphae was studied by growing the cells in RPMI-1640 medium supplemented with 10% FBS (Invitrogen). Different concentrations of SL (0 μg/ml, 15 μg/ml and 30 μg/ml) was added to mature hyphae and incubated for 5 hrs at 37 °C. Subsequently, aliquots of samples were visualized under bright field using 60X objective lens by Zeiss fluorescence microscope and photographed. Samples at time point zero were also examined under similar condition.

### Expression analysis of *Candida albicans* hypha specific genes by qRT-PCR

Effect of sub inhibitory concentration of SL on the expression of hypha specific genes *HWP1*, *ALS1*, *ALS3*, *ECE1* and *SAP4* was evaluated by two-step quantitative real time polymerase chain reaction (qRT-PCR). Total RNA of the cells was extracted from SL treated (15 μg/ml) and untreated (0 μg/ml) hyphal growth samples of RPMI-1640 medium using hot phenol/chloroform extraction method^57^. Following extraction, RNA integrity was assessed on denaturing agarose gel. Thereafter, total RNA was treated with DNase I, amplification grade (Invitogen). cDNA was synthesized from DNase I treated total RNA using iScript™ cDNA Synthesis Kit (BIO-RAD) as per manufacturer’s instructions. Primers for target (*HWP1*, *ALS1*, *ALS3*, *ECE1* and *SAP4*) and housekeeping internal control (*ACT1*) genes were designed using Gene Runner software (Table 3) and synthesized from Sigma. cDNA template (100 ng), gene specific sense and antisense primers (200 nM) and iQ™ SYBR^®^ Green Supermix (BIO-RAD) were used in reaction mixture in accordance with manufacturer’s instructions and qRT-PCR was performed in Mastercycler® ep realplex Real-time PCR system. To check the DNA contamination in templates, DNase I treated total RNA were included in each run. The following parameters were used for qRT-PCR: an initial denaturation at 95 °C (3 min), followed by 40 cycles of denaturation (95 °C/1 min), annealing (58 °C/30 sec), and extension (72 °C/20 sec), melting-curve analysis starting from initial temperature 50 °C to 95 °C, with gradual increase in 0.5 °C/15 second. Specificity of the primers was confirmed by melting curve analysis. The generated C_T_ values of target genes were normalized to the C_T_ value of housekeeping *ACT1* gene. Relative expression fold changes were evaluated by ΔΔC_T_ method using 2^−ΔΔC^_T_ formula^58^.

### Combination testing of SL with AmB and FLZ on *Candida albicans* biofilm formation and preformed biofilm

Nature of the interaction of SL with AmB and FLZ was evaluated by chequerboard assay. Serially double-diluted concentrations of SL (0–1920 μg/ml), AmB (0–32 μg/ml) and FLZ (0–1024 μg/ml) were prepared in RPMI-1640 medium. 50 μl of each dilution of two compounds (SL and AmB or SL and FLZ) were dispensed in 96-well microtiter plates. Biofilm formation was initiated in the presence of combination of compounds and incubated at 37 °C for 2 days as described above. For preformed biofilms, combination of compounds were added into the wells of PBS washed biofilms and further incubated at 37 °C for 2 days. Following incubation, medium was aspirated from the wells and biofilms were washed 3-times with PBS. Thereafter, metabolic activity of biofilms was determined by colorimetric XTT reduction assay. To evaluate the interaction between compounds, Fractional Inhibitory Concentration Index (FICI) was calculated from the data obtained with biological triplicates. FICI is the sum of the FICs of either compound (BIC or BEC _compound A with compound B_/BIC or BEC _compound A_ + BIC or BEC _compound B with compound A_/BIC or BEC _compound B_). The interaction is considered synergistic when the FICI is ≤0.5, indifferent when the value is 0.5 < FICI ≤4 and antagonistic when the value is >4^29^.

### Statistical evaluation

All experiments were performed in triplicate and on three different days. All data were expressed as mean values with the corresponding standard deviations (SD). Statistical significance between treated and control groups was analyzed by Student’s *t*-test (two-tailed, unequal variance). A p-value of <0.05 was considered statistically significant.

## Additional Information

**How to cite this article**: Haque, F. *et al*. Inhibitory Effect of Sophorolipid on *Candida albicans* Biofilm Formation and Hyphal Growth. *Sci. Rep*. **6**, 23575; doi: 10.1038/srep23575 (2016).

## Supplementary Material

Supplementary Information

## Figures and Tables

**Table 1:** List of *Candida* strains used in the SL susceptibility study and their respective MIC_80_, BIC_80_ and BEC_80_ values.

Species and strain	MIC_80_ (μg/ml) of SL	BIC_80_ (μg/ml) of SL	BEC_80_ (μg/ml) of SL
*C. albicans* SC5314^18^	60	120	480
*C. glabrata* CG462^18^	120	480	ND*
*C. tropicalis MYA3404* 50	60	120	480
*C. lusitania*e CL6^18^	30	120	ND*

^*^Not Determined.

**Table 2:** Interaction of sophorolipid with amphotericin B and fluconazole on *C. albicans* biofilm formation and preformed biofilms.

Compound	Biofilm formation		Preformed biofilms	
	FIC^a^	FICI	FIC	FICI
AmBSL	0.2500.125	0.375	0.1250.250	0.375
FLZSL	0.0310.250	0.281	<0.125*0.250	≥0.250 to≤0.375*

^a^FIC was calculated for the concentration at which 80% (BIC_80_ or BEC_80_) reduction in the metabolic activity of biofilm compared to the control.

^*^BEC_80_ of preformed biofilm for FLZ was ≥1024 μg/ml.

**Table 3:** List of primers used for qRT-PCR experiments.

Primer	Sequence (5′-3′)	T_m_(^o^C)	Amplified product size (bp)
CaACT1-RTS	GGTTTGGAAGCTGCTGGTATTGACC	60.8	135
CaACT1-RTAS	ACGTTCAGCAATACCTGGGAACATG	60.0	
CaHWP1-RTS	CAAGTGGTGCTATTACTATTCCG	60.2	121
CaHWP1-RTAS	GCGACACTTGAGTAATTGGC	61.0	
CaALS1-RTS	AGCTGTTGCCAGTGCTTC	60.6	132
CaALS1-RTAS	AATGTGTTGGTTGAAGGTGAG	60.2	
CaALS3-RTS	CAACATCAACCAACCAATCTC	60.6	133
CaALS3-RTAS	TGAATAACAGAACCAGATCCG	60.3	
CaECE1-RTS	CTTCTTCAAAGACTCCCACAAC	60.5	138
CaECE1-RTAS	TTCAATACCGACAGTTTCAATG	60.2	
CaSAP4-RTS	GGTACCGTTGATTTCCAATTC	60.8	132
CaSAP4-RTAS	ATCTTCACTTTCACGAACACG	60.5	
