# Supplementary material for: Inhibitory Effect of Sophorolipid on Candida albicans Biofilm Formation and Hyphal Growth
Source: Sci Rep. 2016 Mar 31;6:23575. doi: 10.1038/srep23575 (PMC4876995; doi:10.1038/srep23575)
Supplement: Supplementary Information [file srep23575-s1.pdf]

Inhibitory Effect of Sophorolipid on *Candida albicans* Biofilm Formation and Hyphal Growth

Running Title: **Effect of Sophorolipid on *Candida albicans* Biofilm**

Farazul Haque[1#], Md. Alfatah[2#], K. Ganesan[2*] & Mani Shankar Bhattacharyya[1*]

[1]Biochemical Engineering Research & Process Development Center (BERPDC), [2]Yeast Molecular Biology Laboratory, CSIR-Institute of Microbial Technology, Sector 39-A, Chandigarh-160 036, India.

[#]Joint first authors

[*]Correspondence should be addressed to: K.G., Phone: +91-172-6665305; Fax: +91-172-2690585; Email: ganesan@imtech.res.in & M.S.B, Phone:+91-172-6665313 Fax: +91-172-2690585; Email: manisb@imtech.res.in.

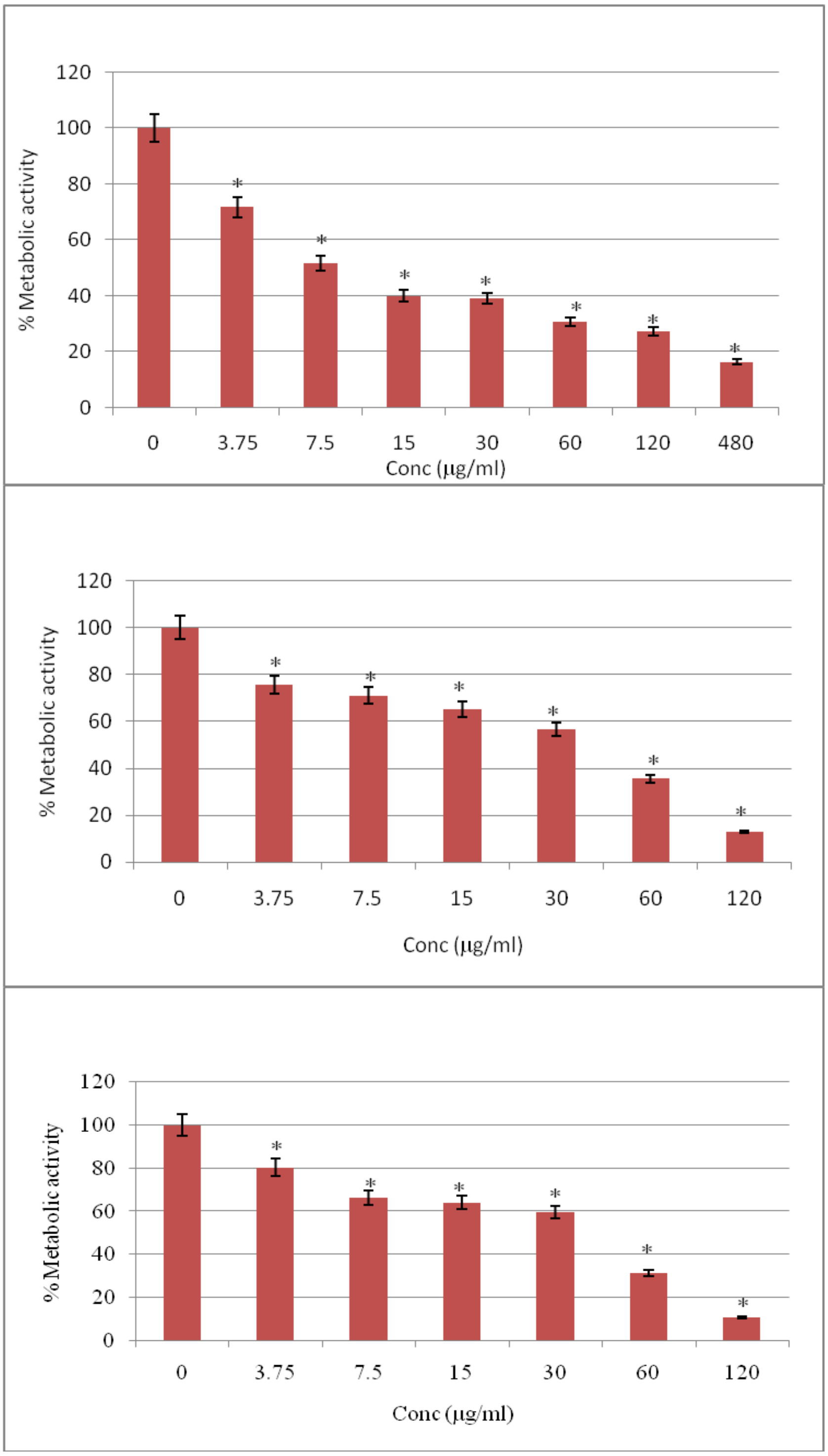

FIG S1 Effect of sophorolipid on (A) *C. glabrata*, (B) *C. tropicalis*, (C) *C. lusitania*e biofilm formation. Readings of colorimetric XTT reduction assay at 492 nm are expressed in terms of % metabolic activity of control. Results represent the average of three independent experiments ± SD. *p<0.05 when compared with the SL untreated controls (0 µg/ml).

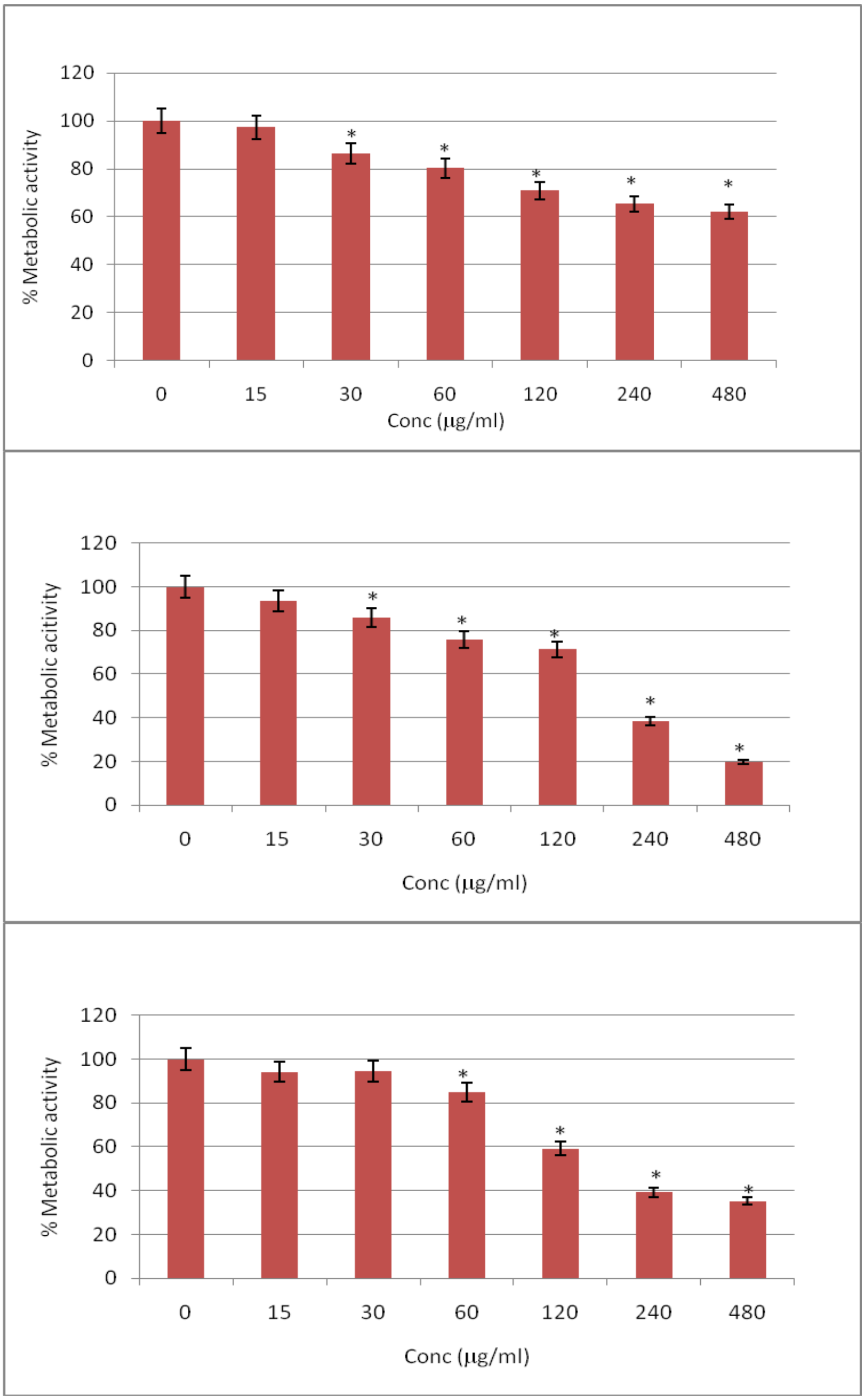

FIG S2 Effect of sophorolipid on (A) *C. glabrata*, (B) *C. tropicalis*, (C) *C. lusitaniae* mature biofilm. Readings of colorimetric XTT reduction assay at 492 nm are expressed in terms of % metabolic activity of control. Results represent the average of three independent experiments ± SD. *p<0.05 when compared with the SL untreated controls (0 µg/ml).

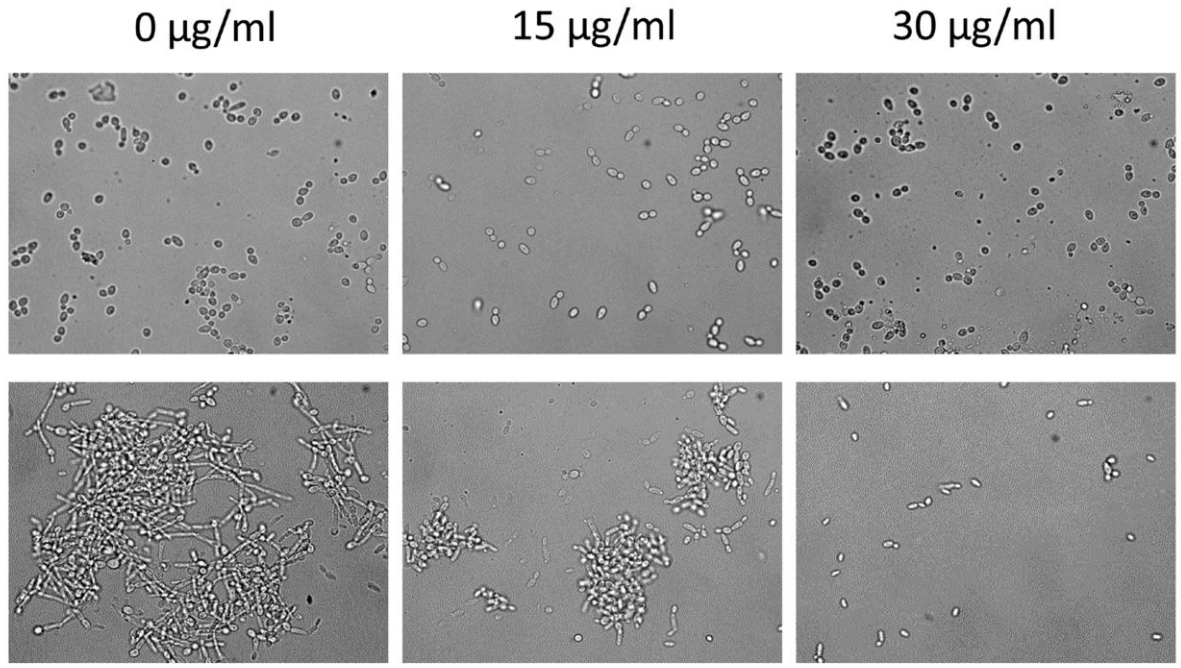

FIG S3 Effect of sophorolipid on *C. albicans* planktonic hyphae. *C. albicans* cells were treated with indicated concentration of SL in RPMI-1640 containing 10% FBS at 37°C for time point zero (upper panel) and 5 hrs (lower panel). At the end of incubation an aliquot was withdrawn from each sample and photographed at 60X magnification

Cultivation of *Starmerella bombicola*, MTCC1910 and SL production

Starmerella bombicola, MTCC1910, was used for the production of SL. *Starmerella bombicola* strain was obtained from MTCC, CSIR- Institute of Microbial Technology, Chandigarh, India. Briefly, the strain was grown in YPD broth at 30°C for 2 days with agitation (200 rpm) for inoculums preparation. 2% of inoculum was added in SL production medium and cultured at 30°C for 7 days in an orbital shaker with agitation (200 rpm). Further cells were separated from the culture broth by centrifugation and extracted three times by ethyl acetate. Ethyl acetate layer was vacuum dried at 40°C and residual hydrophobic components were washed with n-hexanes to give crude SL mixtures.

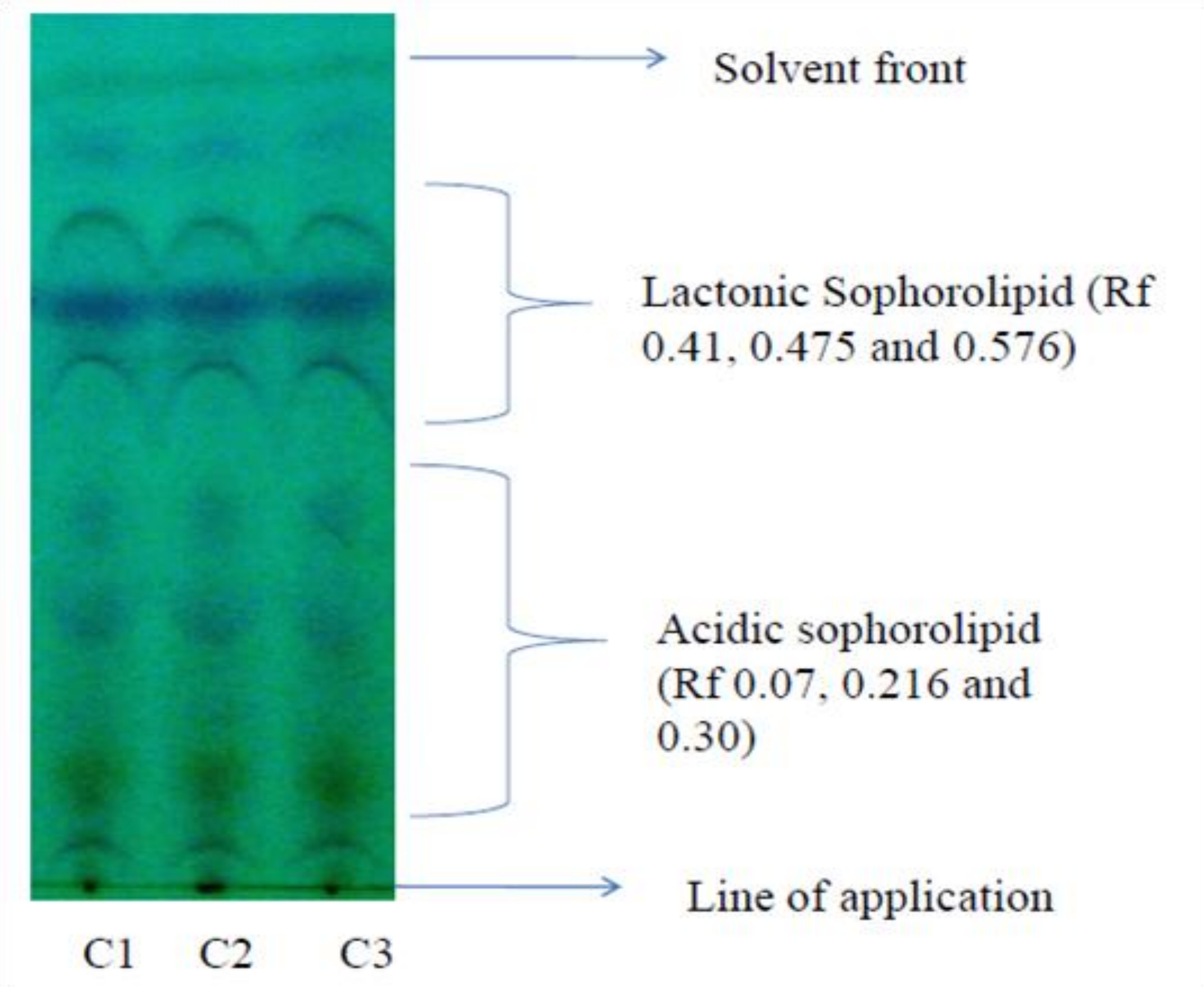

FIG S4 Thin layer chromatogram showing the mixtures of acidic and lactonic form of SL present in the sample produced by *Starmerella bombicola* (MTCC 1910). C1, C2 and C3 indicates the triplicates of crude SL.

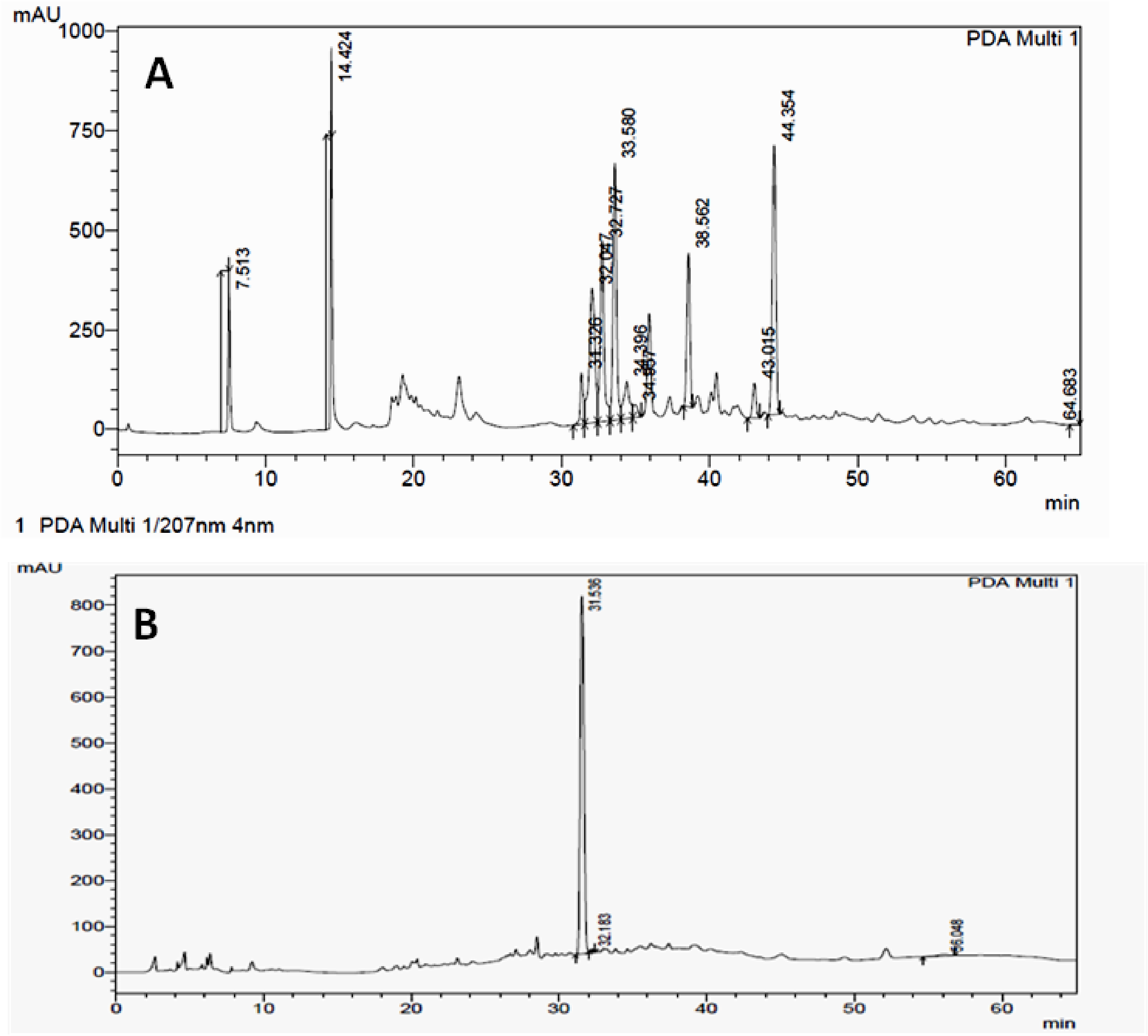

FIG S5 A HPLC chromatogram of the crude SL produced by *Starmerella bombicola* MTCC 1910 (A) and HPLC chromatogram of purified (lactonic) form of SL extracted from the crude mixture (B)

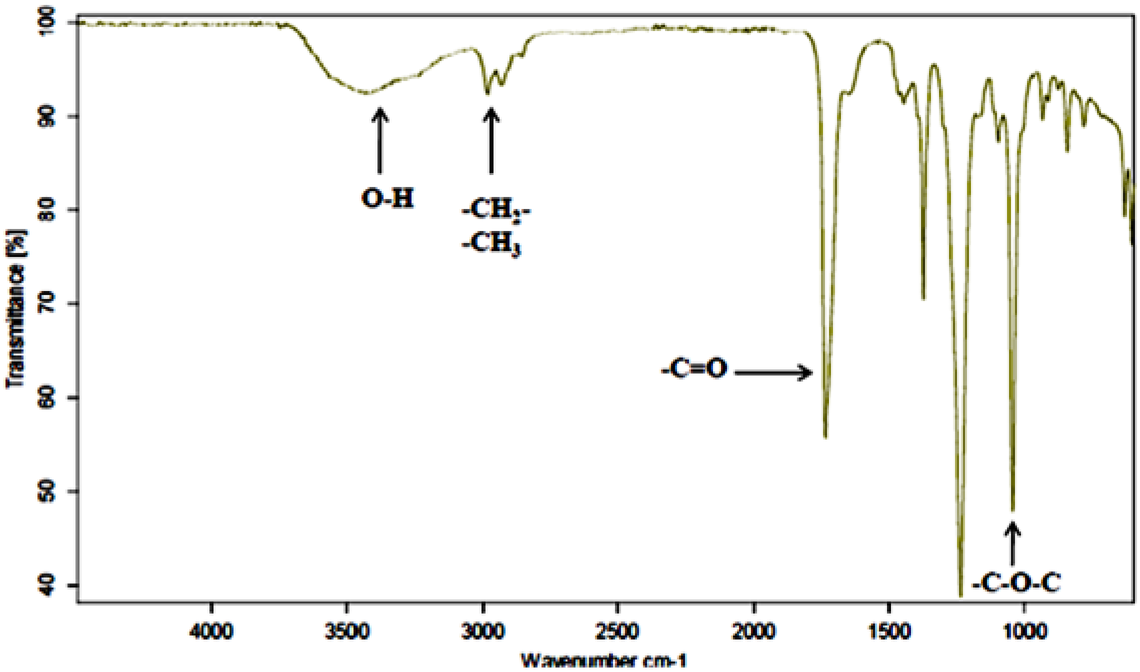

FIG S6 FTIR spectra of crude SL showing presence of acidic and lactonic form.
